# Making the most of malaria chemoprevention

**DOI:** 10.1186/s12936-024-04867-5

**Published:** 2024-02-19

**Authors:** Jasper Littmann, Dorothy Achu, Miriam K. Laufer, Corine Karema, David Schellenberg

**Affiliations:** 1 Bergen Centre for Ethics and Priority Setting—BCEPS, Department of Global Public Health and Primary Care, Faculty of Medicine, University of Bergen, Bergen, Norway; 2 World Health Organization, Regional Office for Africa, Brazzaville, Republic of Congo; 3 Center for Vaccine Development and Global Health, University of Maryland School of Medicine, Baltimore, MD USA; 4 Quality and Equity Healthcare, Kigali, Rwanda; 5 Faculty of Infectious and Tropical Disease, London School of Hygiene and Tropical Medicine, London, UK; 6 Division for Infection Control, The Norwegian Institute for Public Health, Oslo, Norway

**Keywords:** Malaria, Chemoprevention, Seasonal chemoprevention, Policy

## Abstract

Against a backdrop of stalled progress in malaria control, it is surprising that the various forms of malaria chemoprevention are not more widely used. The World Health Organization (WHO) has recommended several malaria chemoprevention strategies, some of them for over a decade, and each with documented efficacy and cost effectiveness. In 2022, the WHO updated and augmented its malaria chemoprevention guidelines to facilitate their wider use. This paper considers new insights into the empirical evidence that supports the broader application of chemoprevention and encourages its application as a default strategy for young children living in moderate to high transmission settings given their high risk of severe disease and death. Chemoprevention is an effective medium-term strategy with potential benefits far outweighing costs. There is a strong argument for urgently increasing malaria chemoprevention in endemic countries.

## Background

After over a decade of advancement in malaria control, the 2016 World Malaria Report (WMR) was a wakeup call, documenting a stagnation in progress [1]. This plateau persists in the 2022 WMR report, with 247 million malaria cases and 619,000 deaths across the globe [2]. The reasons behind the stagnation are complex and contested, but there is widespread agreement that new approaches to prevention are needed.

The pipeline of new tools to control malaria has never been more substantial. In addition to new therapeutic agents, preventive interventions, including improved insecticide-based vector control products, vaccines and monoclonal antibodies (mAbs), and innovative non-pharmaceutical interventions such as genetically modified mosquitoes, are expected to reduce the burden of disease and facilitate malaria control and elimination in the coming decades.

**Fig. 1:**
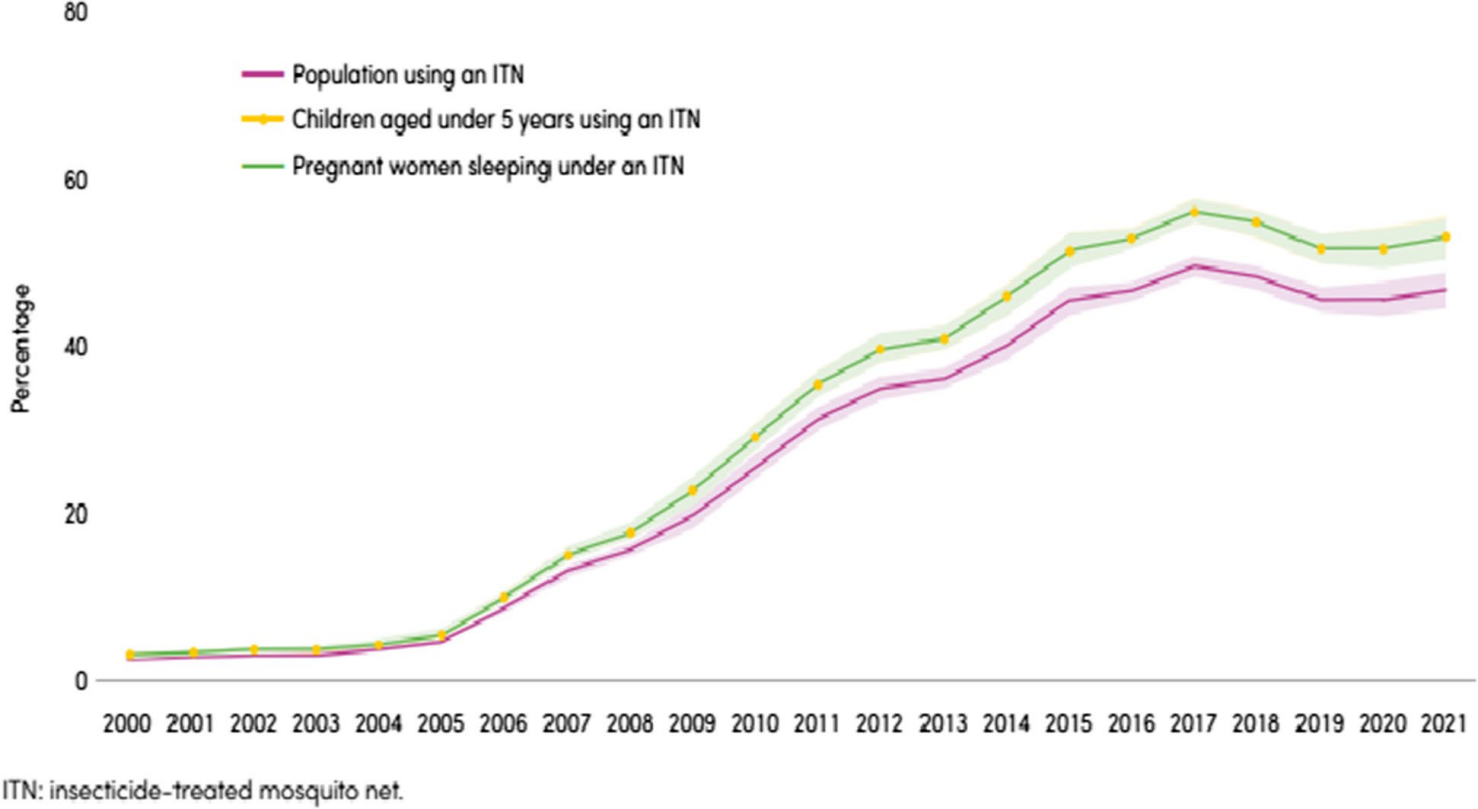
Indicators of population-level use of ITNs, sub-Saharan Africa, 2000–2021 [2]

However, while awaiting new interventions, and given the high, ongoing burden of malaria, available tools must be used to their maximum potential. While some interventions have shown impressive results, there is currently no silver bullet to prevent malaria, as all interventions have challenges that compromise their effectiveness and need to be considered in the context of limited resources. For example, a cornerstone of preventive strategies is vector control. The early part of this century saw massive increases in investments to prevent malaria, including using insecticide-treated mosquito nets (ITN). The proportion of the population sleeping under an ITN increased from 2 to 47% between 2000 and 2021 (Fig. 1). However, sustained investment has struggled to expand coverage further as existing delivery strategies reach the limit of what they can achieve [3] and incur diminishing rates of return because of the difficulties and costs of accessing hard-to-reach populations. Insecticide resistance, human behaviour, vector dynamics, and the physical and chemical durability of mosquito nets compound the coverage challenge. At the same time, chemoprevention is tried and tested but underutilized. It should be possible to save tens or hundreds of thousands of lives every year through better deployment and targeting of existing technologies.

## Preventive use of malaria medicines

Malaria medicines have long been used to prevent malaria in travellers and non-immune people visiting malaria endemic areas. Despite also having been shown to save lives among those living at highest risk (young children) in moderate to high transmission settings [4], malaria medicines have not been widely deployed as a standard intervention for local populations in endemic settings. The reasons for this include five factors:

1. *Relatively high costs of procuring and delivering regular chemoprophylaxis to large numbers of people*
2. *Feasibility of large-scale deployment—the practicalities of accessing a large number of people on a regular basis*
3. *Acceptability and adherence of target groups to repeat dosing*
4. *Potential to accelerate drug resistance which could undermine both the efficacy of the preventive treatment and, if the same drugs are used for treatment, the ability to cure a life-threatening infection.*
5. *Fear of compromising the development or maintenance of naturally acquired immunity, leading to an increase in the risk of malaria among those who had used chemoprophylaxis but then stopped the intervention, the so-called ‘rebound phenomenon’.*

In the last few decades experience has grown in the use of full treatment courses of malaria medicines to prevent disease in endemic settings [5]. This so-called ‘chemoprevention’ is administered at pre-specified times, regardless of the presence of symptoms or infection status, clears any existing infections and prevents new ones for a period. The intermittent nature of most chemoprevention strategies could ameliorate concerns about the costs, feasibility, acceptability, resistance and rebound without losing all the benefits.

### Malaria chemoprevention

The first form of malaria chemoprevention to be recommended by the World Health Organization (WHO), in 1998, was IPT in pregnancy (IPTp). This consists of the administration of a treatment dose of an anti-malarial, usually sulfadoxine-pyrimethamine (SP), at monthly intervals when women attend routine antenatal clinics in the second and third trimesters of pregnancy. This has been shown to reduce maternal anaemia and increase birthweight. The IPTp recommendation was followed, in 2010, by a recommendation for IPT in infants (IPTi, now referred to as perennial malaria chemoprevention—PMC). As its new name suggests, PMC is intended to protect children living in areas with perennial malaria transmission. Initial studies delivered PMC alongside some of the routine vaccinations in the first year of life. In 2012, Seasonal Malaria Chemoprevention (SMC) was recommended to protect children under 5 years old in settings with intensely seasonal malaria transmission [6]. The 2022 WHO World Malaria Report documented that almost 45 million (of an estimated 50 million eligible) children had received SMC, but the coverage of pregnant women receiving three or more doses of IPTp was just 35%, while IPTi had only been implemented in one country [2].

In June 2022 WHO published updates to the chemoprevention recommendations and added three new forms of chemoprevention, namely post-discharge malaria chemoprevention (PDMC), IPT in school children (IPTsc) and Mass Drug Administration (MDA) [7]. The evidence base for each recommendation is summarized in the WHO guidelines. Since the original recommendations (1998–2012), considerable operational experience and new insights into the use of IPTp and SMC have been gathered. The key learnings can be summarized as follows:

### Malaria chemoprevention is affordable

Existing IPTp, PMC and SMC programmes make use of older, less expensive drugs that are no longer used for treatment [7]. Although costs of delivery vary depending on the setting and programme size, chemoprevention is relatively affordable [8], even in comparison with other, highly cost-effective malaria prevention strategies. This is particularly the case when using existing contacts with the health systems to deliver chemoprevention. For example, IPTp delivered through antenatal clinics can be cost-saving in some settings [9] with the cost of delivering individual doses of IPTp-SP ranging from $0.63–$0.79 [10, 11]. Similarly, the cost per dose of PMC-SP [12, 13] delivered through routine immunization contacts was $0.23 per dose [14]. Large funders, such as Unitaid and Givewell, have recognized the potential for wider use of these cost-effectiveness strategies and are supporting large-scale implementation work [15, 16].

### Large-scale deployment of chemoprevention is feasible

Community-based approaches to delivering SMC have seen rollout to ~ 45 m children in 2021, underlining the feasibility of larger scale deployment of malaria chemoprevention in different settings [2]. While this is no proof that deployment will be possible in all settings, it serves to reassure that implementation is possible even where delivery systems have to be developed for the purpose.

### Malaria chemoprevention is acceptable

Success from existing SMC programmes, and from studies of PMC and IPTp, show that acceptance of the interventions is high and that adherence to repeat dosing is satisfactory [12, 13]. For example, one study of over 25 million SMC treatments in over 7.5 million children in a single year documented a mean monthly coverage of 75% with 53% of eligible children treated at all four intended timepoints [17].

### Drug resistance does not undermine the benefits of malaria chemoprevention and is not substantially accelerated by chemoprevention

Existing evidence shows that the effects of chemoprevention on the spread of resistance, and the effects of resistance on chemoprevention, are modest [18]. This is not to say that the risk of emerging drug resistance should be dismissed entirely. Experience with chemoprevention in pregnancy and children shows that, although SP remains effective in areas with very high SP resistance, it is not as effective as in areas with less resistance [12, 13, 19–21]. However, concerns are ameliorated for two additional reasons. Firstly, while drugs that are currently used for chemoprevention were all originally developed for treatment, they are now almost exclusively used for prevention. Chemoprevention thus allows for the continued utilization of older drugs without reducing the effectiveness of newer ones or undermining treatment. Secondly, there is a pipeline of drugs in development for the treatment of malaria and, for the first time, specific consideration of the development of drugs for prevention [22].

### The benefits of chemoprevention outweigh the risks of a rebound effect

There is evidence that a high level of protection from malaria may compromise the development or maintenance of naturally acquired immunity and increase the risk of malaria among those who had used, but then stopped, an intervention. This so-called rebound effect has been repeatedly explored and, although real and measurable, its extent has not outweighed the benefits of the interventions causing it [23]. As more efficacious malaria prevention tools become available there will be a continuing need for vigilance: it is reasonable to assume that rebound will be more pronounced the better people are protected from malaria exposure. Thus, the risk may be enhanced the longer and the more regularly people receive drugs and other interventions. However, the theoretical fear of rebound should not prevent deployment of proven interventions.

### Each dose of a malaria drug provides a period of protection

Each dose of chemoprevention protects the recipient for a specific period of time. The duration of protection varies according to the pharmacokinetics of the drugs and the sensitivity of the parasites in the area concerned. For example, a study of PMC-SP in a setting with no appreciable resistance found that each dose of SP provided protection for about 42 days after treatment [20]. This period was reduced to 21 days in a setting with very high resistance [19]. Nevertheless, even in the high resistance setting, this translated to a 64% reduction in observed cases of malaria during the month following treatment.

Hence, in a perennial transmission setting, a greater number of treatments will protect for a greater proportion of the time at risk. The initial studies of PMC—then called IPTi—evaluated two or four doses of SP between the ages of 2–12 months. As each dose protects for approximately 1 month, IPTi reduced the observed number of cases of malaria by around 30% [24]. Similarly, when three or four rounds of SMC were administered over a 3–4 month transmission season, the intervention reduced the observed number of malaria cases by 72% [25]. Chemoprevention is, therefore, no complete solution but clearly has the potential to deliver meaningful impact. Especially in the perennial transmission settings, the greater the number of doses delivered, the greater the protection that will be provided.

The updated WHO guidelines draw on these lessons to provide flexibility for countries to tailor their chemoprevention strategies to the local malaria epidemiology. The success of SMC should encourage renewed efforts to deliver chemoprevention for the benefit of children living in perennial transmission settings. Initiatives to improve the delivery of vaccines in the 2nd year of life and the availability of Community Health Worker programmes provide opportunities to address existing inequities in access to chemoprevention among those living in perennial and hard to reach settings.

## Ethical implications of the empirical evidence

The process to update the WHO guidelines included careful review of all the evidence for all chemoprevention strategies—a total of 167 studies. This is summarized in the WHO guidelines [5]. Overall, the positive documented effects of chemoprevention strategies confirm they deliver tangible benefits, are acceptable, feasible to deliver and that the financial and non-financial costs are limited, both to the recipient (side effects, rebound effect) and to society (emergence of drug resistance). This led to the formulation of guidelines which are more permissive, providing flexibility to national malaria programmes to tailor chemoprevention strategies to the local settings. There is now an opportunity to broaden malaria chemoprevention, recognizing also that the pipeline of new drugs and other preventive interventions is relatively healthy, mitigating the effect of any emerging drug resistance in the medium term, and that completely new preventative strategies (including monoclonal antibodies, multistage malaria vaccines, gene-drive mosquitoes) are being developed. Together these may limit the period for which chemoprevention is a necessary part of malaria control efforts, further limiting the costs associated with long-term use of the strategies.

Based on the available empirical evidence, and the expected costs and benefits of chemoprevention to individuals and society, there is not just an economic and medical, but also an ethical case for the broader application of chemoprevention. To reduce the burden of malaria in line with the Sustainable Development Goals, it will be necessary to roll out malaria chemoprevention programmes widely and quickly. From a consequentialist perspective, if an intervention is a cost-effective way of saving lives, and funds for its implementation can be made available, its introduction should only be delayed if (i) more cost-effective interventions are not yet implemented, or (ii) wider societal concerns (such as costs to future generations) count so heavily against it that they influence the overall cost–benefit calculation. The insights outlined above reassure that any wider societal concerns are unlikely to be realized in practice, which means that if condition (i) is met, there is a strong normative reason for implementing chemoprevention programmes. Chemoprevention strategies are relatively inexpensive and among the most cost-effective malaria prevention tools. Even if the costs associated with the intervention were higher, this would be insufficient grounds for dismissing it. Instead, it would have to be shown that the costs exceed the expected benefits.

### Framework for decision-making

The available empirical evidence points towards an overall positive cost–benefit balance for chemoprevention as an intermediate intervention to reduce childhood morbidity and mortality. However, the concerns about chemoprevention merit consideration by control programs and others. Decisions about implementation will have always to be made in relation to local realities. There may be a tipping point at which the potential costs of the intervention outweigh its benefits in some situations. It is, therefore, essential to consider local and regional characteristics that may influence impact and costs and to whom the various costs and benefits will accrue. For malaria chemoprevention, benefits and costs can be identified at both the individual and societal levels, in the short term and for future generations. Although not exhaustive, Table 1 offers a framework to consider these issues. When considering, implementation of malaria chemoprevention, five relevant considerations should be met, in addition to two more general points about the use of cost–benefit analysis.

**Table 1:**
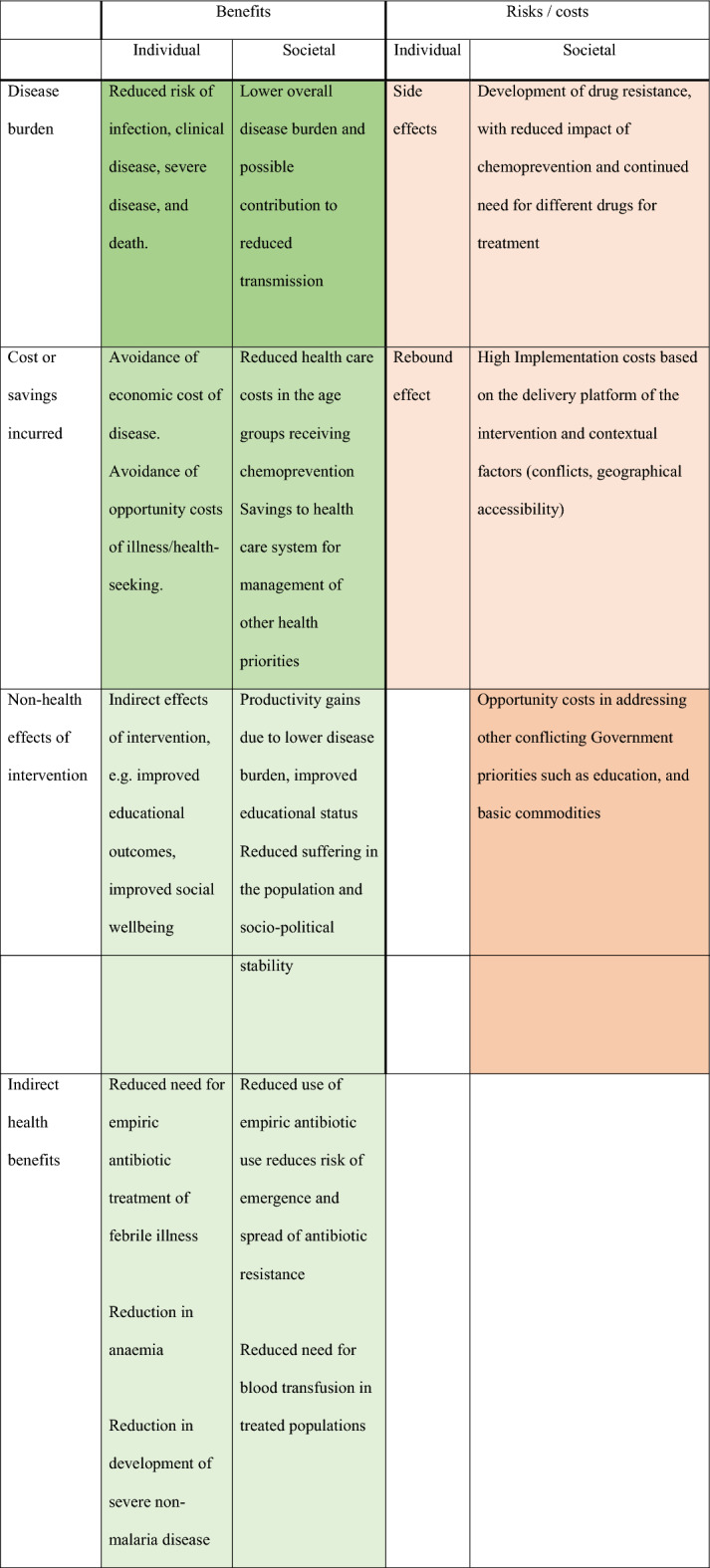
Selected benefits and risks associated with the roll out of chemoprevention: Colour indicates the positive and negative effect sizes (e.g. deeper shade of green indicates a greater positive effect)

Effect sizes are the authors’ estimates. Colours represent the effect size, with deeper shades of green / orange representing greater risks and costs respectively

### There is reasonable certainty that chemoprevention will have positive health effects

Available evidence suggests that each dose of chemoprevention will protect for a defined period [19–21] and thus, especially in perennial transmission settings, the more doses received, the greater the benefit. The reduced morbidity resulting from chemoprevention will likely translate into improved survival in younger age groups.

### Adverse health outcomes of the intervention are very limited

The known side effects of drugs used for chemoprevention are limited and severe adverse effects are rare and almost always outweighed by the reductions in morbidity and mortality for individuals and society [7].

### Effect on drug resistance appears limited

The preventive use of some antimicrobials may be discouraged because resultant resistance would undermine the therapeutic efficacy of the drug for future recipients. This concern has been raised with the potential mass drug administration of azithromycin [26]. However, to date, the drugs used for malaria chemoprevention are generally not the same as those used for treatment, given the availability of more efficacious alternatives, and cross-resistance between drug classes is not a major concern. Moreover, malaria chemoprevention may help slow the emergence of antibiotic resistance if it reduces the use of antibiotic treatment for febrile illness.

### Alternative drugs are available for prevention and treatment

The pipeline for new drugs and vaccines against malaria is better now than ever before. This reduces the risk of having to use drugs presently used for chemoprevention for treatment in the future. Furthermore, efforts are now being made to develop drugs specifically for malaria prevention [22].

### Financial costs, cost-effectiveness, and opportunity costs of chemoprevention

Given the high disease burden and low costs of chemoprevention, it is likely to be cost-effective in most malaria endemic settings. The level of cost-effectiveness will depend on the disease burden in the setting and details of the intervention, including choice of drug, delivery strategy, size of the target group, and the geography of the implementation site. As with the introduction of any new intervention, opportunity costs should be considered: (a) could money that is spent on chemoprevention achieve an even more significant impact if it were used differently, and (b) will funding for chemoprevention come at the expense of ongoing interventions: if so, will this diversion in funds result in an overall improvement of expected health outcomes?

Other approaches to malaria prevention (e.g. vector control, vaccines) are not mutually exclusive and can combine with chemoprevention to form high impact intervention packages [27].

### To whom do the costs and benefits of the intervention accrue?

*If some of the costs are likely borne by others (including future persons), how are these weighted against present-day benefits?*

While it is likely that future generations will partly bear both costs and benefits, it is difficult to quantify:

- a) what share of costs and benefits will be borne by whom, and.
- b) whether costs and benefits are spread out evenly over time (i.e. if future generations will incur a net benefit or net costs over time).

Although the empirical evidence is reassuring, the most likely non-financial cost may be some form of drug-resistance that limits the options for chemoprevention in the future [18]. Potential benefits will first and foremost accrue to the individuals who avoid infection and experience less morbidity and mortality. The wide roll-out of chemoprevention will likely have additional positive long-term effects, including increased productivity and lower demand for health care services. These societal benefits are recognized, but their effect size is difficult to measure and heavily dependent on the setting [28].

### Discount rate: how long into the future should we consider potential costs/benefits?

Any discount rate will depend on:

- a) how big the impact on future generations is expected to be, and.
- b) how long it would take to mitigate the negative effects of an intervention.

Overall, the negative effects of chemoprevention are expected to be limited for future generations. Mitigation strategies would mainly require the shift to a different drug for chemoprevention, which may have been developed specifically for the purpose. It seems unlikely that the interests of future generations are sufficiently negatively affected by the intervention to be counted against it.

## Conclusion

Malaria chemoprevention is underutilized. The historic concerns which hindered the more widespread use of chemoprevention are now known not to outweigh the potential benefits at both personal and societal levels in the short and long term. Chemoprevention is not a long-term solution nor a stand-alone strategy to address malaria. However, until better forms of malaria prevention are sufficiently widely available to render chemoprevention superfluous, there are medical, epidemiological, economic, and ethical arguments in favour of its expanded use.

## Abbreviations

IPT: Intermittent preventive treatment
IPTi: Intermittent preventive treatment in infants
IPTp: Intermittent preventive treatment in pregnancy
IPTsc: Intermittent preventive treatment in school children
ITN: Insecticide-treated mosquito net
PDMC: Post-discharge malaria chemoprevention
PMC: Perennial malaria chemoprevention
PMC-SP: Perennial malaria chemoprevention—Sulfadoxine-pyrimethamine
mABS: Monoclonal antibodies
MDA: Mass drug administration
SMC: Seasonal malaria chemoprevention
SP: Sulfadoxine-pyrimethamine

## References

[1] WHO (2016). World malaria report 2016.

[2] WHO (2022). World malaria report 2022.

[3] Bhatt S, Weiss DJ, Mappin B, Dalrymple U, Cameron E, Bisanzio D (2015). Coverage and system efficiencies of insecticide-treated nets in Africa from 2000 to 2017. Elife.

[4] Greenwood BM, Greenwood AM, Bradley AK, Snow RW, Byass P, Hayes RJ (1988). Comparison of two strategies for control of malaria within a primary health care programme in the Gambia. Lancet.

[5] World Health Organization. 2022. Updated WHO recommendations for malaria chemoprevention among children and pregnant women. https://www.who.int/news/item/03-06-2022-Updated-WHO-recommendations-for-malaria-chemoprevention-among-children-and-pregnant-women Accessed 04 May 2023

[6] WHO (2012). Policy recommendation: seasonal malaria chemoprevention (SMC) for *Plasmodium falciparum* malaria control in highly seasonal transmission areas of the Sahel sub-region in Africa.

[7] WHO (2022). Guidelines for malaria.

[8] Conteh L, Shuford K, Agboraw E, Kont M, Kolaczinski J, Patouillard E (2021). Costs and cost-effectiveness of malaria control interventions: a systematic literature review. Value Health.

[9] Sicuri E, Bardaji A, Nhampossa T, Maixenchs M, Nhacolo A, Nhalungo D (2010). Cost-effectiveness of intermittent preventive treatment of malaria in pregnancy in southern Mozambique. PLoS ONE.

[10] Fernandes S, Sicuri E, Kayentao K, van Eijk AM, Hill J, Webster J (2015). Cost-effectiveness of two versus three or more doses of intermittent preventive treatment for malaria during pregnancy in sub-Saharan Africa: a modelling study of meta-analysis and cost data. Lancet Global Health.

[11] Hansen KS, Ndyomugyenyi R, Magnussen P, Clarke SE (2012). Cost-effectiveness analysis of three health interventions to prevent malaria in pregnancy in an area of low transmission in Uganda. Int Health.

[12] Rodriguez E, Ahn J, van Eijk A, Gutman J. Contextual factors influencing intermittent preventive treatment in pregnancy with sulfadoxine-pyrimethamine (IPTp-SP) uptake. Zenodo. 2022. 10.5281/zenodo.6559913

[13] Steinhardt L. Summary of intermittent preventive treatment in infants (IPTi) contextual factors. Zenodo. 2022. 10.5281/zenodo.6535570

[14] Manzi F, Hutton G, Schellenberg J (2008). From strategy development to routine implementation: the cost of intermittent preventive treatment in infants for malaria control. BMC Health Serv Res.

[15] Unitaid. A new initiative will deliver enhanced malaria prevention to children under two across Africa through a WHO-recommended but under-implemented intervention. 2021. https://unitaid.org/news-blog/ipti-enhanced-malaria-prevention-for-children/#en Accessed 05 May 2023

[16] Givewell. Malaria Consortium—Seasonal Malaria Chemoprevention. 2023. https://www.givewell.org/charities/malaria-consortium. Accessed 05 May 2023

[17] ACCESS-SMC Partnership (2020). Effectiveness of seasonal malaria chemoprevention at scale in west and central Africa: an observational study. Lancet.

[18] Plowe CV (2022). Malaria chemoprevention and drug resistance: a review of the literature and policy implications. Malar J.

[19] Cairns M, Gosling R, Carneiro I, Gesase S, Mosha JF, Hashim R (2010). Duration of protection against clinical malaria provided by three regimens of intermittent preventive treatment in Tanzanian infants. PLoS ONE.

[20] Cairns M, Carneiro I, Milligan P, Owusu-Agyei S, Awine T, Gosling R (2008). Duration of protection against malaria and anaemia provided by intermittent preventive treatment in infants in Navrongo. Ghana PLoS ONE.

[21] Griffin JT, Cairns M, Ghani AC, Roper C, Schellenberg D, Carneiro I (2010). Protective efficacy of intermittent preventive treatment of malaria in infants (IPTi) using sulfadoxine-pyrimethamine and parasite resistance. PLoS ONE.

[22] Medicines for Malaria Venture. Pipeline of antimalarial drugs. 2022. https://www.mmv.org/research-development/mmvs-pipeline-antimalarial-drugs. Accessed 03 May 2023

[23] WHO (2022). Technical consultation on the malaria rebound phenomenon: report on a virtual meeting.

[24] Aponte JJ, Schellenberg D, Egan A, Breckenridge A, Carneiro I, Critchley J (2009). Efficacy and safety of intermittent preventive treatment with sulfadoxine-pyrimethamine for malaria in African infants: a pooled analysis of six randomised, placebo-controlled trials. Lancet.

[25] Thwing JI, Williamson J, Cavros I, Bhattarai A, Gutman J. Seasonal malaria chemoprevention for malaria in children in areas with seasonal malaria. Zenodo. 2022. 10.5281/zenodo.6535576

[26] WHO (2020). Guideline on mass drug administration of azithromycin to children under five years of age to promote child survival.

[27] Chandramohan D, Zongo I, Sagara I, Cairns M, Yerbanga RS, Diarra M (2021). Seasonal malaria vaccination with or without seasonal malaria chemoprevention. N Engl J Med.

[28] Andrade MV, Noronha K, Diniz BPC, Guedes G, Carvalho LR, Silva VA (2022). The economic burden of malaria: a systematic review. Malar J.

